# Supplementary material for: The three stages of religious decline around the world
Source: Nat Commun. 2025 Aug 19;16:7202. doi: 10.1038/s41467-025-62452-z (PMC12365078; doi:10.1038/s41467-025-62452-z)
Supplement: Supplementary file 1 — Supplementary Information [file 41467_2025_62452_MOESM1_ESM.pdf]

The Three Stages of Religious Decline Around the World. Supplementary Information

Jörg Stolz, Nan Dirk de Graaf, Conrad Hackett, Jean-Philippe Antonietti

SUPPLEMENTARY METHODS ... 2
1. The PIB sequence and continent ... 2
2. The PIB sequence and historical religion ... 4
3. Robustness against including/excluding controls ... 5
4. Robustness when controlling for gender ... 6
5. The PIB sequence and cultural zone ... 7
6. Fit per country ... 8
7. Robustness against different specifications of country secularity ... 12
8. Robustness against different specifications of private religiosity ... 14
9. Robustness with respect to the function (logit vs. probit) ... 16
10. Robustness against other cut-off points for cohorts ... 17
11. Robustness against other cut-off points for religiosity indicators ... 17
12. Robustness against including/excluding imputation of missing values, weights ... 18
13. Robustness with respect to different prior specifications ... 19
14. Finding theoretical time ... 20
SUPPLEMENTARY DISCUSSION ... 21
1. A formal model of the link between modernization and the PIB sequence ... 21
2. HDI and cohort gaps in religiosity ... 23
REFERENCES ... 24

SUPPLEMENTARY METHODS

1. The PIB sequence and continent

The PIB sequence can be perceived on all continents. Africa, however, only shows the first two stages (Fig. 1). Posterior midpoints are given in Table 1. The CI's (significance tests) are given in Table 2.

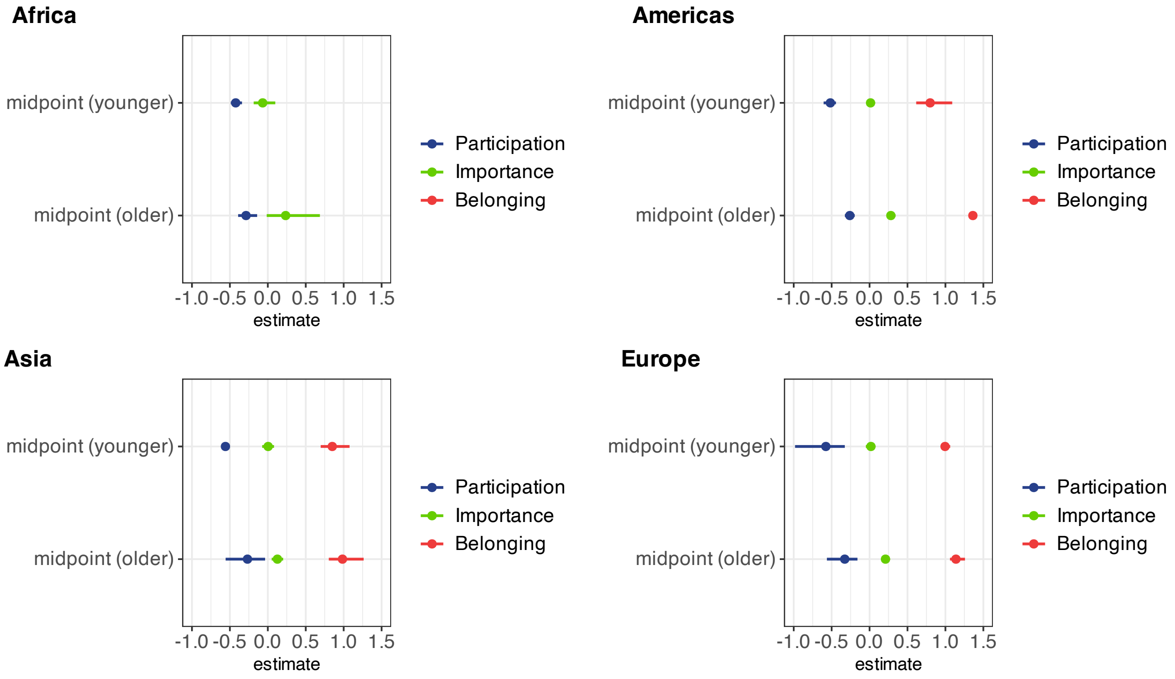

Fig. 1. Coefficients of midpoints in the cross-sectional models for participation, importance of religion, and belonging for different continents

Notes: Pew data. N(Africa) = 24; N(Americas) = 21; N(Asia) = 32; N(Europe) = 34. We visualize the midpoints for older (40+) and younger (<40) individuals for the sigmoid models predicting percentage of weekly participation, high importance of religion, and religious belonging. The lines represent highest (posterior) density intervals at the 95% level. Since the models are not linear, the intervals do not have to be symmetric. We do not control for other variables.

Table 1 Results for different continents

	Partici-pation	Impor-tance	Belonging	Partici-pation	Impor-tance	Belonging
	Africa			Americas		
Midpoint older	-0.29 (0.06)	0.23 (0.19)	5.55 (5.39)	-0.26 (0.03)	0.28 (0.03)	1.36 (0.71)
Midpoint younger	-0.42 (0.04)	-0.07 (0.07)	6.02 (5.59)	-0.52 (0.04)	0.01 (0.02)	0.80 (0.12)
	Asia			Europe		
Midpoint older	-0.27 (0.13)	0.13 (0.04)	0.98 (0.12)	-0.33 (0.10)	0.21 (0.02)	1.14 (0.05)
Midpoint younger	-0.56 (0.51)	0.00 (0.04)	0.85 (0.10)	-0.58 (0.18)	0.02 (0.03)	1.00 (0.03)

Note: We report posterior midpoints (standard errors).

Table 2 Credibility intervals for differences in different continents

	Difference	CI (95%)
Africa:		
Difference younger-older: participation	-0.136	-0.298; -0.002
Difference younger-older: importance	-0.303	-0.768; -0.001
Difference younger-older: belonging	0.47	-15.588;16.453
Difference younger: participation - importance	-0.355	-0.533; -0.212
Difference younger: participation - belonging	-6.445	-20.636; -0.542
Difference younger: importance - belonging	-6.089	-20.275; -0.192
Difference older: participation - importance	-0.522	-0.982; -0.229
Difference older: participation - belonging	-5.839	-19.888; -0.466
Difference older: importance - belonging	-5.317	-19.423; 0.082
Americas:		
Difference younger - older: participation	-0.256	-0.366; -0.157
Difference younger - older: importance	-0.268	-0.343; -0.197
Difference younger - older: belonging	-0.563	-2.308; 0.095
Difference younger: participation - importance	-0.53	- 0.628; -0.443
Difference younger: participation - belonging	-1.316	-1.614; -1.114
Difference younger: importance - belonging	-0.786	-1.077; -0.595
Difference older: participation - importance	-0.541	-0.631; -0.455
Difference older: participation - belonging	-1.622	-3.36; -1.052
Difference older: importance - belonging	-1.081	-2.808; -0.511
Asia		
Difference younger - older: participation	-0.29	-0.792; 0.136
Difference younger - older: importance	-0.122	-0.232; -0.014
Difference younger - older: belonging	-0.135	-0.457; 0.157
Difference younger: participation - importance	-0.563	-1.028; -0.253
Difference younger: participation - belonging	-1.408	-1.907; -1.050
Difference younger: importance - belonging	-0.845	-1.082; -0.672
Difference older: participation - importance	-0.395	-0.689; -0.149
Difference older: participation - belonging	-1.253	-1.655; -0.944
Difference older: importance - belonging	-0.858	-1.147; -0.660
Europe		
Difference younger - older: participation	-0.249	-0.685; 0.104
Difference younger - older: importance	-0.192	-0.271; -0.116
Difference younger - older: belonging	-0.144	-0.274; -0.04
Difference younger: participation - importance	-0.594	-1.004; -0.334
Difference younger: participation - belonging	-1.571	-1.979; -1.313
Difference younger: importance - belonging	-0.977	-1.07; -0.895
Difference older: participation - importance	-0.537	-0.777; -0.362
Difference older: participation - belonging	-1.466	-1.723; -1.274
Difference older: importance - belonging	-0.929	-1.056; -0.837

Note: Pew data. Significance tests using highest (posterior) density intervals, 95%. The first three rows test within the indicators whether younger cohorts are significantly less religious than older cohorts regarding participation, importance, and belonging. The next three rows show test whether the midpoints between indicators for the younger cohorts are significantly different. The last three rows do the same of the older cohorts.

2. The PIB sequence and historical religion

The PIB sequence can be perceived in historically Muslim, Buddhist and Hindu, as well as Christian countries (Fig. 2). The posterior midpoints are given in Table 3. The CI's (significance tests) are given in Table 4. In historically Christian countries, the full sequence can be observed. In historically Muslim countries, only the two first steps can be observed, since there are not (yet?) strongly secularized historically Muslim countries. In historically Buddhist/Hindu countries, the estimations for participation are to be interpreted with caution since participation was measured differently in different surveys.

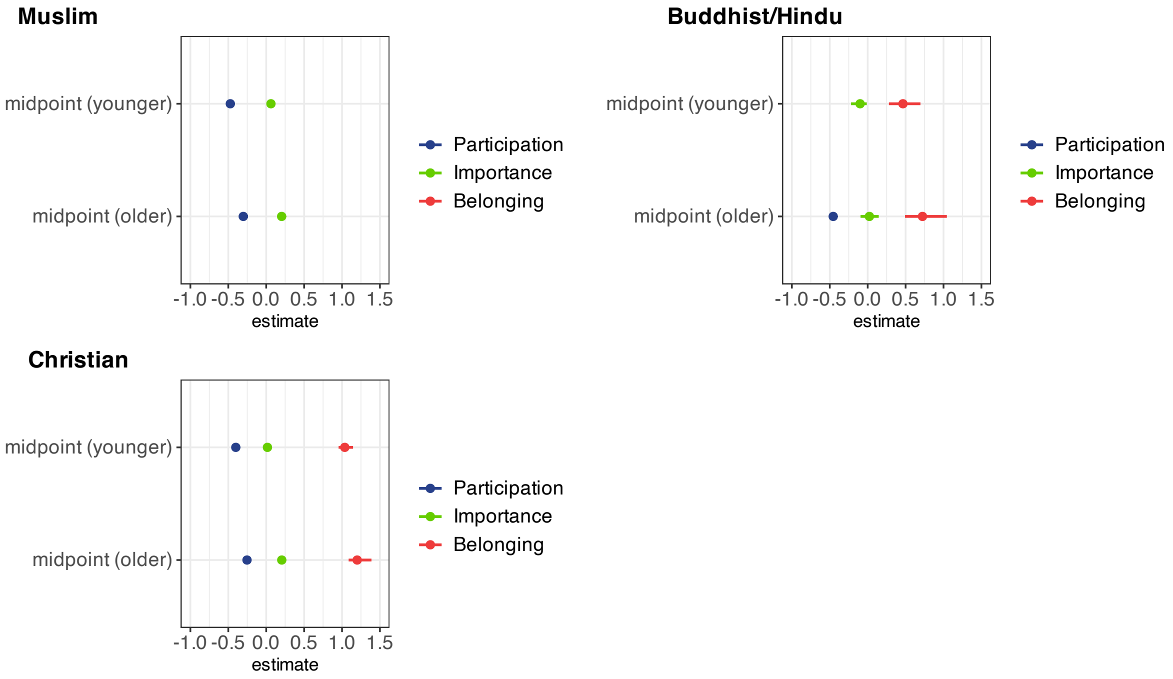

Fig. 2 Coefficients of midpoints in the cross-sectional models for participation, importance of religion, and belonging for countries with different historical religions

Notes: Pew data. N(Muslim) = 28; N(Buddhist/Hindu) = 11; N(Christian) = 71. We do not show traditional religion = Jewish (N = 1). We visualize the midpoints for older (40+) and younger (<40) individuals for the sigmoid models predicting percentage of weekly participation, high importance of religion, and religious belonging. The lines represent highest (posterior) density intervals at the 95% level. Since the models are not linear, the intervals do not have to be symmetric. We do not control for other variables. Confidence intervals for participation in historically Buddhist/Hindu countries are not shown for better visibility.

Table 3 Results for different religious traditions

	Muslim			Buddhist/Hindu		
	Partici-pation	Impor-tance	Belonging	Partici-pation	Impor-tance	Belonging
Midpoint older	-0.30 (0.03)	0.20 (0.03)	7.58 (5.36)	-0.46 (3.74)	0.02 (0.06)	0.72 (0.14)
Midpoint younger	-0.47 (0.03)	0.06 (0.03)	8.00 (5.46)	-1.71 (3.37)	-0.10 (0.05)	0.47 (0.10)
	Christian					
Midpoint older	-0.25 (0.02)	0.20 (0.01)	1.20 (0.08)			
Midpoint younger	-0.40 (0.02)	0.02 (0.01)	1.04 (0.05)			

Note: We report posterior midpoints (standard errors).

Table 4 Credibility intervals for differences in different religions

	Difference	CI (95%)
Muslims:		
Difference younger-older: participation	-0.171	-0.254; -0.09
Difference younger-older: importance	-0.141	-0.218; -0.067
Difference younger-older: belonging	0.415	-15.592; 16.027
Difference younger: participation - importance	-0.536	-0.612; -0.46
Difference younger: participation - belonging	-8.471	-22.274; -1.982
Difference younger: importance - belonging	-7.935	-21.758; -1.44
Difference older: participation - importance	-0.506	-0.586; -0.424
Difference older: participation - belonging	-7.885	-21.588; -1.91
Difference older: importance - belonging	-7.379	-21.083; -1.413
Buddhist/Hindu:		
Difference younger - older: participation	-1.257	-13.24; 9.58
Difference younger - older: importance	-0.123	-0.289; 0.028
Difference younger - older: belonging	-0.258	-0.628; 0.074
Difference younger: participation - importance	-1.612	-11.459; 1.471
Difference younger: participation - belonging	-2.177	-12.048; 0.902
Difference younger: importance - belonging	-0.565	-0.824; -0.359
Difference older: participation - importance	-0.477	-10.151; 6.968
Difference older: participation - belonging	-1.178	-10.86; 6.249
Difference older: importance - belonging	-0.700	-1.045; -0.441
Christian		
Difference younger - older: participation	-0.148	-0.21; -0.084
Difference younger - older: importance	-0.189	-0.231; -0.147
Difference younger - older: belonging	-0.164	-0.367; -0.003
Difference younger: participation - importance	-0.417	-0.47; -0.366
Difference younger: participation - belonging	-1.437	-1.553; -1.344
Difference younger: importance - belonging	-1.02	- 1.132; -0.932
Difference older: participation - importance	-0.458	-0.514; -0.404
Difference older: participation - belonging	-1.453	-1.646; -1.331
Difference older: importance - belonging	-0.995	-1.186; -0.878

Note: Pew data. Significance tests using highest (posterior) density intervals, 95%. The first three rows test within the indicators whether younger cohorts are significantly less religious than older cohorts regarding participation, importance, and belonging. The next three rows show test whether the midpoints between indicators for the younger cohorts are significantly different. The last three rows do the same of the older cohorts.

3. Robustness against including/excluding controls

To investigate whether our results are robust against using or not using the controls, we ran the model with Pew Data and WVS7 data without controls. The coefficients are given in Table 5. The coefficients are very similar. The CI's (significance tests) are given in Table 6.

Table 5 Results with and without controls

	(1) baseline (with controls)			(2) without controls		
	Partici-pation	Impor-tance	Belonging	Partici-pation	Impor-tance	Belonging
Pew						

Midpoint older	-0.37 (0.04)	0.01 (0.03)	1.01 (0.06)	-0.22 (0.03)	0.17 (0.02)	1.17 (0.06)
Midpoint younger	-0.53 (0.04)	-0.15 (0.03)	0.81 (0.05)	-0.40 (0.03)	0.00 (0.02)	1.01 (0.05)
WVS/EVS7						
Midpoint older	-0.48 (0.07)	0.08 (0.04)	0.96 (0.05)	-0.49 (0.05)	0.03 (0.02)	0.90 (0.04)
Midpoint younger	-0.68 (0.07)	-0.06 (0.04)	0.76 (0.05)	-0.68 (0.06	-0.11 (0.02)	0.72 (0.04)

Note: We report posterior midpoints (standard errors).

Table 6 Credibility intervals for differences without controls

	Difference	CI (95%)
Pew:		
Difference younger-older: participation	-0.175	-0.261; -0.088
Difference younger-older: importance	-0.176	-0.22; -0.132
Difference younger-older: belonging	-0.156	-0.316; -0.017
Difference younger: participation - importance	-0.399	-0.466; -0.331
Difference younger: participation - belonging	-1.412	-1.524; -1.312
Difference younger: importance - belonging	-1.013	-1.115; -0.93
Difference older: participation - importance	-0.399	-0.469; -0.33
Difference older: participation - belonging	-1.393	-1.544; -1.273
Difference older: importance - belonging	-0.994	-1.138; -0.888
WVS/EVS7:		
Difference younger - older: participation	-0.197	-0.349; -0.047
Difference younger - older: importance	-0.141	-0.19; -0.093
Difference younger - older: belonging	-0.187	-0.295; -0.084
Difference younger: participation - importance	-0.57	-0.691; -0.463
Difference younger: participation - belonging	-1.401	-1.538; -1.279
Difference younger: importance - belonging	-0.831	-0.911; -0.756
Difference older: participation - importance	-0.515	-0.629; -0.411
Difference older: participation - belonging	-1.392	-1.526; -1.268
Difference older: importance - belonging	-0.877	-0.966; -0.795

Note: Pew and WVS/EVS7 data. Significance tests using highest (posterior) density intervals, 95%. The first three rows test within the indicators whether younger cohorts are significantly less religious than older cohorts regarding participation, importance, and belonging. The next three rows show test whether the midpoints between indicators for the younger cohorts are significantly different. The last three rows do the same of the older cohorts.

4. Robustness when controlling for gender

Our study uses gender as a control variable in the WVS7 data, where the variable is available. We find that the P-I-B sequence is robust to controlling for gender. In Table 7 we give the results separately for women and men. We again see both the expected differences between the P-I-B indicators and the expected differences between older and younger cohorts. The CI's are given in Table 8.

Table 7 Results for women and men

	Women			Men		
	Partici-pation	Impor-tance	Belonging	Partici-pation	Impor-tance	Belonging

WVS/EVS7						
Midpoint older	-0.5 (0.07)	0.07 (0.02)	0.87 (0.04)	-0.46 (0.04)	0.01 (0.02)	0.90 (0.05)
Midpoint younger	-0.71 (0.07)	-0.07 (0.02)	0.7 (0.03)	-0.64 (0.04)	-0.12 (0.02)	0.71 (0.04)

Note: We report posterior midpoints (standard errors).

Table 8 Credibility intervals for differences for women and men

Women	Difference	CI (95%)
Difference younger-older: participation	-0.209	-0.402; -0.021
Difference younger-older: importance	-0.141	-0.199; -0.084
Difference younger-older: belonging	-0.171	-0.269; -0.077
Difference younger: participation - importance	-0.637	-0.791; -0.507
Difference younger: participation - belonging	-1.407	-1.571; -1.264
Difference younger: importance - belonging	-0.77	-0.847; -0.695
Difference older: participation - importance	-0.569	-0.714; -0.44
Difference older: participation - belonging	-1.369	-1.523; -1.228
Difference older: importance - belonging	-0.799	-0.885; -0.721
Men		
Difference younger-older: participation	-0.184	-0.306; -0.064
Difference younger-older: importance	-0.136	-0.198; -0.074
Difference younger-older: belonging	-0.192	-0.323; -0.069
Difference younger: participation - importance	-0.520	-0.621; -0.428
Difference younger: participation - belonging	-1.356	-1.48; -1.244
Difference younger: importance - belonging	-0.836	-0.931; -0.748
Difference older: participation - importance	-0.472	-0.571; -0.378
Difference older: participation - belonging	-1.364	-1.50; -1.242
Difference older: importance - belonging	-0.891	-01.005; -0.792

Note: Pew and WVS/EVS7 data. Significance tests using highest (posterior) density intervals, 95%. The first three rows test within the indicators whether younger cohorts are significantly less religious than older cohorts regarding participation, importance, and belonging. The next three rows show test whether the midpoints between indicators for the younger cohorts are significantly different. The last three rows do the same of the older cohorts.

5. The PIB sequence and cultural zone

We do not adjust for cultural zone in our final model. It is, however, instructive to inspect just where countries of different cultural zones find themselves according to the PIB sequence. This is what Fig. 3 shows.

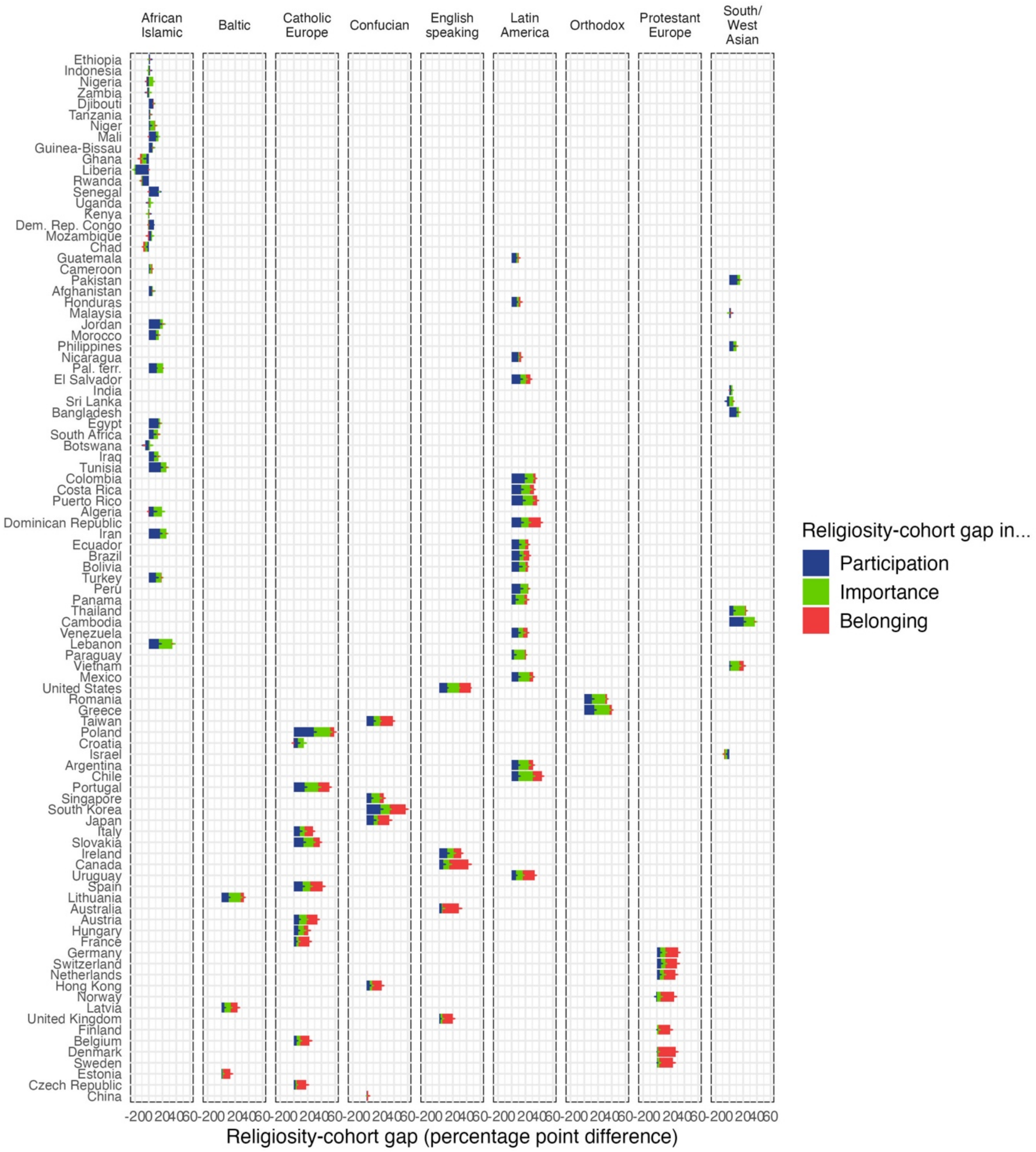

Fig. 3 Religiosity-cohort gap in participation, importance, and belonging in different countries and cultural zones (country secularity ranked)

Note: Pew Data. Countries are ordered according to country secularity with lower country secularity on top and higher country secularity on the bottom. We plot the 95% confidence intervals. For better visibility, only half of the confidence interval is plotted - for positive percentage values at the right of the respective percentage bar, for negative percentage values at the left of the respective percentage bar.

6. Fit per country

We calculated four measures of fit for every country, across indicators and for older and younger cohorts. Since we are focused on differences between older and younger generations, we are not only interested in the absolute deviation for every country, but also in the deviation delta for every country, that is, a deviation measure for the difference between

the predicted and the real difference between the older and the younger cohort in that country across indicators. These indicators are calculated as follows (and displayed in Table 5)

The positive deviation:

$$\text{for } y_{ijk} > m_{ijk}: \quad \text{dev.pos(i)} = \sum_{1}^{ijk} \left(\frac{y_{ijk} - m_{ijk}}{s_{jk}}\right)^2 \tag{1}$$

Where y is the true value and m is the predicted value. s stands for the standard deviation. Subscript i denotes the country, j the indicator and k a dichotomous variable older/younger.

The negative deviation:

$$\text{for } y_{ijk} < m_{ijk}: \quad \text{dev.neg(i)} = \sum_{1}^{ijk} \left(\frac{y_{ijk} - m_{ijk}}{s_{jk}}\right)^2 \tag{2}$$

The absolute deviation:

$$\text{dev.abs(i)} = \text{abs(dev.pos(i))} + \text{abs(dev.neg(i))} \tag{3}$$

The deviation delta:

$$\text{dev.delta(i)} = \text{dev.pos(i)} - \text{dev.neg(i)} \tag{4}$$

Table 9 Deviations per country (Pew data)

**Country**	**dev.pos**	**dev.neg**	**dev.abs**	**dev.delta**
Vietnam	38.58	59.20	97.77	-20.62
South Korea	22.07	32.95	55.02	-10.88
United States	0.83	7.34	8.17	-6.51
Taiwan	42.43	48.62	91.05	-6.19
Hong Kong	4.14	9.80	13.94	-5.65
Czech Republic	0.10	5.03	5.13	-4.94
Dominican Republic	0.80	5.55	6.34	-4.75
Egypt	0.36	4.32	4.68	-3.96
Canada	2.00	5.58	7.59	-3.58
Mexico	1.40	4.95	6.35	-3.55
Greece	1.36	4.68	6.04	-3.32
Poland	4.54	7.65	12.20	-3.11
Norway	1.03	3.65	4.68	-2.61
Netherlands	2.06	4.55	6.62	-2.49
Puerto Rico	0.04	2.46	2.50	-2.43
Turkey	0.02	2.36	2.38	-2.34
China	0.02	2.27	2.29	-2.26
Botswana	1.18	3.34	4.52	-2.16
El Salvador	0.21	2.33	2.54	-2.12
Romania	0.33	2.34	2.67	-2.01
Honduras	0.14	1.87	2.01	-1.73
Tunisia	0.06	1.55	1.61	-1.50
Venezuela	1.36	2.82	4.18	-1.46

Costa Rica	0.13	1.59	1.73	-1.46
Bosnia-Herzegovina	1.09	2.35	3.44	-1.26
Singapore	1.79	2.95	4.74	-1.15
Brazil	0.09	1.17	1.27	-1.08
Colombia	0.13	1.20	1.33	-1.07
Algeria	0.05	1.05	1.09	-1.00
Chad	0.76	1.75	2.51	-0.99
Panama	0.48	1.46	1.93	-0.98
Mozambique	2.40	3.37	5.77	-0.97
Italy	0.39	1.36	1.75	-0.96
Sweden	0.03	0.97	1.00	-0.94
Morocco	0.73	1.59	2.32	-0.86
Thailand	0.89	1.72	2.61	-0.84
Spain	0.72	1.53	2.25	-0.82
Nicaragua	0.68	1.49	2.17	-0.81
South Africa	0.09	0.88	0.97	-0.79
India	0.27	1.01	1.28	-0.75
Portugal	0.86	1.60	2.46	-0.73
Iraq	1.01	1.72	2.73	-0.71
Slovakia	0.97	1.63	2.59	-0.66
Iran	1.05	1.70	2.75	-0.65
Ecuador	1.02	1.62	2.64	-0.60
Croatia	0.13	0.71	0.84	-0.58
Uganda	0.86	1.41	2.27	-0.54
Malaysia	0.07	0.53	0.60	-0.47
Peru	0.89	1.35	2.24	-0.46
Ghana	1.28	1.73	3.02	-0.45
Germany	0.49	0.93	1.42	-0.45
Belgium	0.03	0.37	0.40	-0.35
Guatemala	0.30	0.63	0.94	-0.33
France	0.14	0.45	0.60	-0.31
Bolivia	0.12	0.43	0.55	-0.31
Switzerland	1.08	1.37	2.46	-0.29
Jordan	0.24	0.43	0.67	-0.20
Palestinian territories	0.54	0.65	1.19	-0.10
Kenya	1.04	1.08	2.11	-0.04
Afghanistan	0.65	0.61	1.26	0.04
Liberia	1.22	1.18	2.40	0.04
Bangladesh	0.20	0.11	0.31	0.09
Cameroon	0.16	0.05	0.21	0.11
Hungary	0.70	0.53	1.23	0.18
Austria	1.49	1.24	2.73	0.25
Moldova	1.82	1.53	3.35	0.29
Lebanon	0.84	0.50	1.34	0.34
Democratic Republic of the Congo	1.03	0.65	1.68	0.38
Paraguay	0.62	0.22	0.84	0.40
Australia	2.52	2.07	4.58	0.45
Japan	21.77	21.31	43.08	0.47

Nigeria	1.64	1.15	2.80	0.49
Rwanda	0.90	0.41	1.31	0.49
Ireland	0.56	0.07	0.62	0.49
Estonia	0.98	0.46	1.44	0.51
Pakistan	1.42	0.87	2.29	0.55
Tanzania	0.68	0.03	0.71	0.65
Uruguay	4.50	3.85	8.35	0.66
Serbia	2.88	2.22	5.10	0.66
Sri Lanka	5.69	4.90	10.59	0.79
Ethiopia	0.82	0.00	0.82	0.82
Guinea-Bissau	0.98	0.15	1.14	0.83
Mali	0.84	0.00	0.84	0.84
Latvia	1.93	1.08	3.02	0.85
Tajikistan	0.91	0.01	0.92	0.90
Kosovo	1.93	0.94	2.87	0.99
Indonesia	1.04	0.00	1.04	1.04
Niger	2.66	1.58	4.24	1.08
Philippines	2.13	1.00	3.13	1.13
Zambia	1.33	0.19	1.52	1.14
Djibouti	2.07	0.76	2.83	1.32
Senegal	2.08	0.48	2.56	1.59
Denmark	2.21	0.60	2.81	1.61
United Kingdom	2.19	0.38	2.57	1.81
Lithuania	4.82	2.53	7.35	2.29
Finland	3.05	0.57	3.61	2.48
Kyrgyzstan	3.23	0.67	3.90	2.56
Argentina	2.85	0.25	3.11	2.60
Ukraine	2.73	0.07	2.81	2.66
Chile	3.64	0.89	4.53	2.75
Belarus	3.79	0.86	4.65	2.93
Cambodia	4.90	1.54	6.44	3.36
Bulgaria	4.64	0.89	5.52	3.75
Israel	4.46	0.58	5.03	3.88
Kazakhstan	4.28	0.15	4.43	4.14
Russia	4.76	0.24	5.00	4.52
Armenia	10.38	3.78	14.17	6.60
Georgia	8.63	1.79	10.42	6.84
Uzbekistan	7.53	0.68	8.21	6.85
Azerbaijan	12.83	3.31	16.14	9.52
Albania	12.40	0.63	13.02	11.77

The results visualized in Fig. 4.

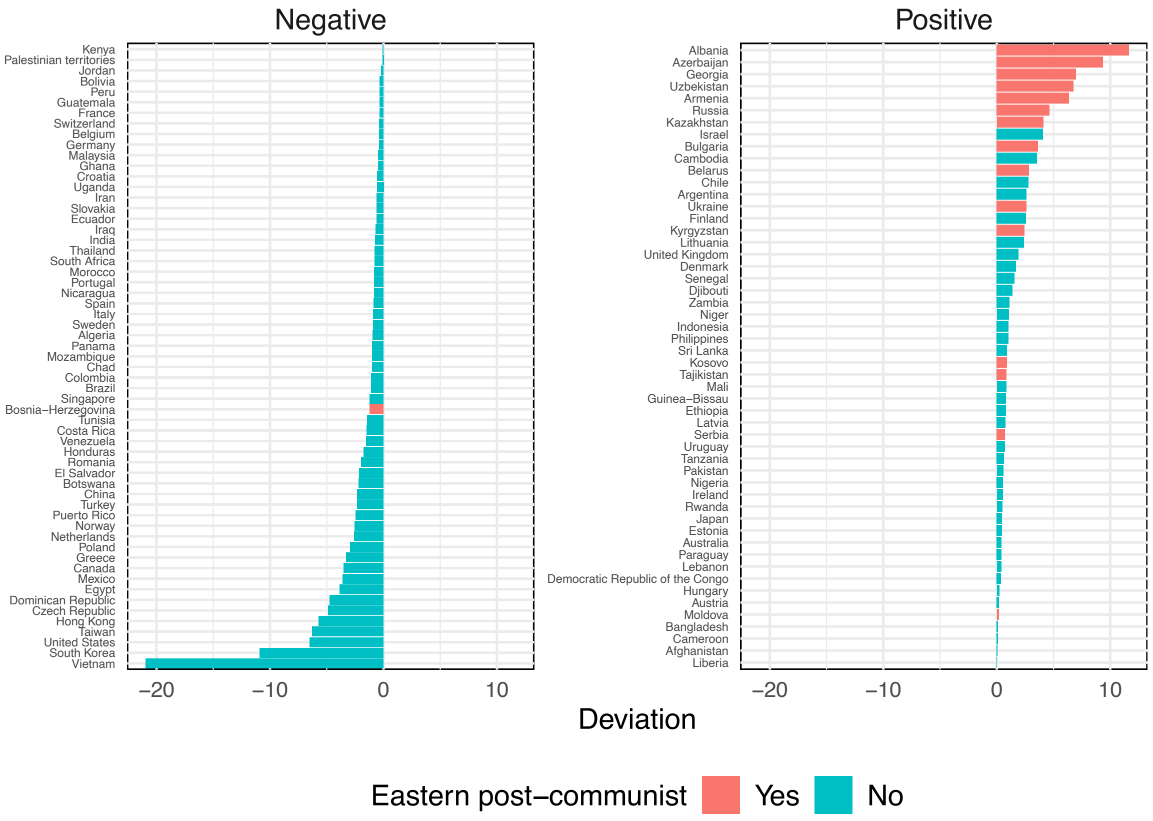

Fig. 4 Deviation delta for different countries (ordered)
Note: Pew data. Deviation delta ordered from largest (top) to smallest (bottom). Deviation delta is defined as dev.delta(i) = dev.pos(i) - dev.neg(i).

7. Robustness against different specifications of country secularity

To investigate whether our results are robust against varying the way country secularity (CS) is operationalized, we check three additional ways of constructing country secularity and test whether the models differ significantly from our initial model (using CS1). We conduct these tests with WVS/EVS7 data, since CS3 and CS4 can only be calculated for this dataset.

It turns out that our results are robust against changing the way country secularity is constructed (Table 10).

CS1: participation, importance of religion, belonging (baseline)
CS2: participation, importance of religion, prayer, belonging
CS3: participation, importance of religion, importance of god, prayer, belonging
CS4: participation, importance of religion, importance of god, prayer, belief in god, religious person, belonging

Table 10 Results with different ways of operationalizing country secularity

	CS1 (baseline)			CS2		
	Partici-pation	Impor-tance	Belonging	Partici-pation	Impor-tance	Belonging
WVS/EVS7						
Midpoint older	-0.49 (0.05)	0.03 (0.02)	0.91 (0.04)	-0.50 (0.07)	0.07 (0.02)	0.87 (0.04)

Midpoint younger	-0.68 (0.05)	-0.11 (0.02)	0.72 (0.04)	-0.71 (0.06)	-0.08 (0.02)	0.73 (0.04)
	CS3			CS4		
	Partici-pation	Impor-tance	Belonging	Partici-pation	Impor-tance	Belonging
WVS/EVS7						
Midpoint older	-0.50 (0.06)	0.05 (0.02)	0.90 (0.04)	-0.50 (0.06)	0.02 (0.02)	0.95 (0.05)
Midpoint younger	-0.71 (0.06)	-0.10 (0.02)	0.75 (0.04)	-0.69 (0.06)	-0.13 (0.02)	0.76 (0.04)

Note: WVS/EVS7 data. We report posterior midpoints (standard errors).

The CI's (significance tests) are given in Table 11.

Table 11 Credibility intervals for differences for different ways of operationalizing country secularity

CS2	Difference	CI (95%)
Difference younger-older: participation	-0.212	-0.39; -0.041
Difference younger-older: importance	-0.145	-0.201; -0.088
Difference younger-older: belonging	-0.158	-0.269; -0.052
Difference younger: participation - importance	-0.632	-0.776; -0.507
Difference younger: participation - belonging	-1.441	-1.597; -1.3
Difference younger: importance - belonging	-0.808	-0.893; -0.726
Difference older: participation - importance	-0.565	-0.695; -0.443
Difference older: participation - belonging	-1.386	-1.533; -1.251
Difference older: importance - belonging	-0.821	-0.915; -0.739
CS3		
Difference younger - older: participation	-0.206	-0.385; -0.033
Difference younger - older: importance	-0.153	-0.208; -0.098
Difference younger - older: belonging	-0.154	-0.265; -0.049
Difference younger: participation - importance	-0.607	-0.75; -0.486
Difference younger: participation - belonging	-1.456	-1.613; -1.318
Difference younger: importance - belonging	-0.849	-0.936; -0.767
Difference older: participation - importance	-0.554	-0.687; -0.434
Difference older: participation - belonging	-1.404	-1.556; -1.271
Difference older: importance - belonging	-0.85	-0.943; -0.768
CS4		
Difference younger - older: participation	-0.192	-0.353; -0.038
Difference younger - older: importance	-0.155	-0.218; -0.092
Difference younger - older: belonging	-0.184	-0.311; -0.061
Difference younger: participation - importance	-0.561	-0.692; -0.448
Difference younger: participation - belonging	-1.458	-1.606; -1.325
Difference younger: importance - belonging	-0.898	-0.993; -0.807
Difference older: participation - importance	-0.523	-0.648; -0.407
Difference older: participation - belonging	-1.45	1.601; -1.313
Difference older: importance - belonging	-0.927	-1.035; -0.829

Note: WVS/EVS7 data. Significance tests using highest (posterior) density intervals, 95%. The first three rows test within the indicators whether younger cohorts are significantly less religious than older cohorts regarding participation, importance, and belonging. The next three rows show test whether the midpoints between

indicators for the younger cohorts are significantly different. The last three rows do the same of the older cohorts.

8. Robustness against different specifications of private religiosity

Our concept of importance points to one dimension of private religiosity. This concept could also be measured with several other indicators - or with a composite measure. Other possible measures of such private religiosity include frequency of prayer, belief in god, describing oneself as a religious person, importance of god, or confidence in religious institutions.

We have decided to use only one measure - importance of religion - for two reasons. On the one hand, this measure has been shown to be the most reliable and comparable measure of overall religiosity (when excluding participation and belonging) [1]. On the other hand, studies on measurement invariance that rely on multi-indicator measurements have shown that it is quite difficult to find a well-fitting measurement model for a large number of countries.

In what follows, we test whether and how results would change when using different indicators for private religiosity. Our robustness tests are different for the Pew and the WVS/EVS7 data. In the aggregated Pew data set we only have one additional indicator: frequency of prayer. In the WVS/EVS7 dataset, we can use frequency of prayer, belief in god, describing oneself as a religious person, importance of god, and confidence in religious institutions.

These indicators were measured as follows:

Prayer was measured in (very slightly) differing wordings in the Pew surveys. The most common wording was: People practice their religion in different ways. Outside of participating in religious services, do you pray several times a day, once a day, a few times a week, once a week, a few times a month, seldom, or never? In the WVS/EVS prayer was measured with the question Apart from weddings and funerals, about how often do you pray? Response options were several times a day, once a day, several times each week, only when participating in religious services, only on special holy days, once a year, less often, never, practically never.

Importance of religion was measured, in the Pew surveys, with the question: How important is religion in your life – very important, somewhat important, not too important, or not at all important? In the WVS/EVS, importance of religion is measures with the question: For each of the following, indicate how important it is in your life. Would you say it is: very important, rather important, not very important, not at all important. The respondents reacted to the Item: Religion.

Importance of god was only used in WVS/EVS. It was measured on a scale from 1 (Not at all important) to 10 (Very important).

Belief in god was only used in WVS/EVS. It was asked as In which of the following things do you believe, if you believe in any? - God? It was coded as 1 (Yes) and 0 (No).

Self-description as a religious person was measured with the item: Independently of whether you go to church or not, would you say you are... Response options were: a religious person, not a religious person, an atheist..

Both for Pew and WVS/EVS surveys, we transformed all indicators of religiosity into dichotomous variable with 1 = weekly attendance, and 0 otherwise; 1 = seeing religion as very important in one's life, and 0 otherwise; 1 = giving the importance of god a 10 on a 1-10 rating scale, and 0 otherwise; 1 = being affiliated with a religion, and 0 otherwise, 1 = describing oneself as a religious person and 0 otherwise; 1 = having a great deal of confidence in the church and 0 otherwise. We then calculated the percentage of individuals per country with the respective religiosity indicator. Table 12 presents the results. The results for Private religiosity I are the same as those for importance of religion in the main paper. The columns Private religiosity II and Private religiosity III show what happens when we use composite measures for private religiosity.

Private religiosity I: importance of religion (baseline)
Private religiosity II: composite measure: importance of religion + prayer.
Private religiosity III: composite measure: importance of religion, importance of god, belief in god, self-description as a religious person.

It turns out that composite variables give us somewhat different midpoints than if we used the single indicator importance of religion. However, these midpoints are always significantly higher than midpoints of Participation and lower than those of Belonging.

Table 12 Results for different ways of measuring Private religiosity

	Private religiosity I (baseline)	Private religiosity II	Private religiosity III
Pew			
Midpoint older	0.01	-0.02	-
	(0.03)	(0.04)	-
Midpoint younger	-0.15	-0.23	-
	(0.03)	(0.04)	-
WVS/EVS 7			
Midpoint older	0.04	0.07	0.07
	(0.04)	(0.05)	(0.05)
Midpoint younger	-0.09	-0.10	-0.12
	(0.04)	(0.05)	(0.05)

Note: We report posterior midpoints (standard errors).

The CI's (significance tests) are given in Table 13.

Table 13 Credibility intervals for differences for different ways of measuring private religiosity

	Difference	CI (95%)
Private religiosity II		
Pew: Difference younger-older	-0.204	-0.255; -0.154
WVS/EVS7: Difference younger-older	-0.167	-0.236; -0.099
Private religiosity III		

WVS/EVS7: Difference younger - older:	-0.185	-0.272; -0.101

Note: WVS/EVS7 data. Significance tests using highest (posterior) density intervals, 95%. The first three rows test within the indicators whether younger cohorts are significantly less religious than older cohorts regarding participation, importance, and belonging. The next three rows show test whether the midpoints between indicators for the younger cohorts are significantly different. The last three rows do the same of the older cohorts.

9. Robustness with respect to the function (logit vs. probit)

In the main paper we use sigmoid functions (inverse logit) to model our three dependent variables. We chose the logit function due to its more intuitive interpretability compared to alternative link functions. However, one might question whether the results would differ under alternative specifications, for example the probit function. The probit function is often used when it is assumed that y is obtained by thresholding a continuous latent variable. In our case, the binary variables were created by using thresholds. Non-parametric functions are not feasible because of the relatively small number of countries. Fig. 5 gives an estimation of participation, importance, and belonging, for older and younger groups, as well as their difference, for the Pew data. The results look very similar to what we saw with a logit function. Table 14 shows that the AIC of logit and probit results is also very similar.

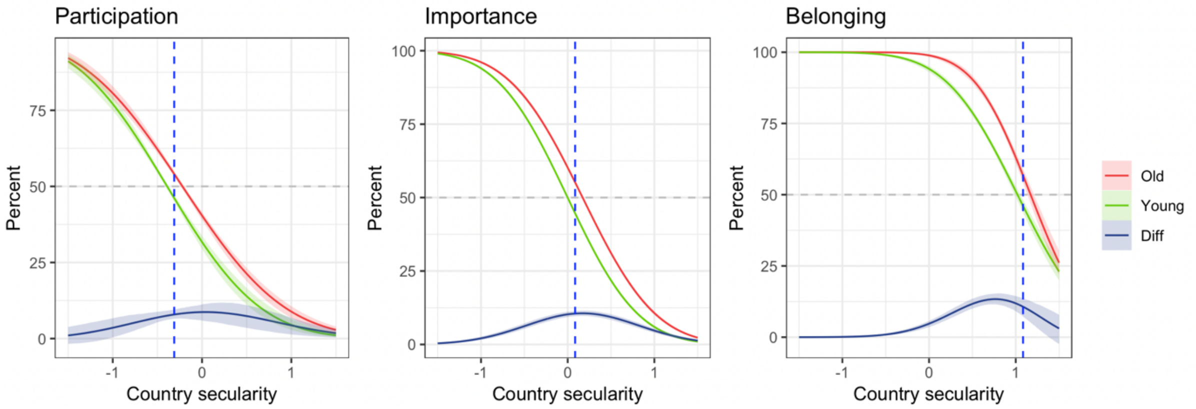

Fig. 5 Probit models of participation, importance, and belonging for older and younger and difference older-younger

Note: Pew data. All countries. Diff = difference between older and younger cohorts. The Figure shows the rise and fall of participation-, importance-, and belonging differences between older and younger individuals in one graph. We show 50% error bands (credible intervals) around the lines. The dotted vertical lines show the midpoints of the estimated functions for participation, importance, and belonging.

Table 14 AIC of logit vs. probit models

	Logit	Probit
Participation	1726.75	1726.93
Importance	1502.62	1504.89
Belonging	1719.29	1725.28

In conclusion, results remain very similar when using a logit or a probit function.

10. Robustness against other cut-off points for cohorts

In our study, the cut-off point used to construct an older and a younger cohort in the Pew and the WVS/EVS7 data sets is 40. Our results are robust with respect to using other cut-off points

This can be demonstrated following the strategy used in Pew[2] in showing that the percentage differences between cohorts are very stable against changing the cut-offs (Table 15). Changing the cutoff point between older and younger results in no more than a 2 percentage-point difference in the average size of the cohort gap.

Table 15 Percentage points by which younger adults are less religious with each age cutoff in the average country

	Age cutoff at ______ years old				
	30	35	40	45	50
Pew Data					
Participation	5	6	6	6	7
Importance	6	6	6	6	7
Belonging	5	5	5	5	5
WVS/EVS7					
Participation	6	7	7	8	8
Importance	6	6	6	6	6
Belonging	6	6	6	6	6

Note: Source: For the Pew Data, we report the values given in Pew 2. The WVS/EVS7 values stem from our own calculation.

11. Robustness against other cut-off points for religiosity indicators

We also checked what average difference between the older and younger cohort we obtain when using a different cut-point for participation and importance (Table 16). Belonging is dichotomous, so there is no robustness test necessary. For participation, we check two different cut-points, for importance only one different cut-point (since the variable is a 4-step variable).

For example, in our main analysis we look at differences between older and younger cohorts regarding individuals who attend weekly (or not). The cut point is at the highest category and the percentage difference is 6. When we change the cut-point to include the next category (monthly religious attendance), the average percentage difference between older and younger cohort is 7.

Table 16 Percentage points by which younger adults are less religious

	highest category = 1	highest and next category = 1	highest and next two categories = 1
WVS/EVS7			
Participation	6	7	6
Importance	5	6	-

The result of this test is that percentage differences remain very similar when varying the cut-points of the religiosity indicators.

12. Robustness against including/excluding imputation of missing values, weights

To further investigate the robustness of our results, we conduct our analysis
(1) with imputed missing values, weights, controls (baseline as in paper)
(2) without the imputation of missing values
(3) without weights.

Since the Pew analysis is conducted on an aggregated dataset that is already weighted, we conduct our robustness analysis only on the WVS data to see the influence of using or not using the weights (Table 17).

We find that our results are robust against these alternative specifications.

Table 17 Results without imputed missing values, without weights

	(1) without imputed missing values			(2) without weights		
	Partici-pation	Impor-tance	Belonging	Partici-pation	Impor-tance	Belonging
Pew						
Midpoint older	-0.37 (0.04)	0.02 (0.03)	1.01 (0.06)	-	-	-
Midpoint younger	-0.53 (0.04)	-0.15 (0.03)	0.81 (0.05)	-	-	-
WVS/EVS7						
Midpoint older	-0.49 (0.07)	0.08 (0.04)	0.96 (0.05)	-0.49 (0.07)	0.09 (0.04)	0.97 (0.05)
Midpoint younger	-0.68 (0.07)	-0.04 (0.04)	0.77 (0.05)	-0.68 (0.07)	-0.04 (0.04)	0.77 (0.04)

Note: We report posterior midpoints (standard errors).

The CI's (significance tests) are given in Table 18.

Table 18 Credibility intervals for differences for different ways of measuring private religiosity

Without imputed missing values (Pew)	Difference	CI (95%)
Difference younger-older: participation	-0.162	-0.252; -0.072
Difference younger-older: importance	-0.164	-0.203; -0.125
Difference younger-older: belonging	-0.196	-0.325; -0.08
Difference younger: participation - importance	-0.383	-0.453; -0.315
Difference younger: participation - belonging	-1.346	-1.456; -1.244
Difference younger: importance - belonging	-0.962	-1.051; -0.884
Difference older: participation - importance	-0.386	-0.459; -0.313
Difference older: participation - belonging	-1.38	-1.52; -1.256
Difference older: importance - belonging	-0.994	-1.117; -0.892
Without imputed missing values (WVS/EVS7)		
Difference younger-older: participation	-0.192	-0.354; -0.036
Difference younger-older: importance	-0.128	-0.176; -0.08

Difference younger-older: belonging	-0.199	-0.31; -0.092
Difference younger: participation - importance	-0.636	-0.779; -0.514
Difference younger: participation - belonging	-1.447	-1.608; -1.303
Difference younger: importance - belonging	-0.81	-0.896; -0.728
Difference older: participation - importance	-0.572	-0.703; -0.455
Difference older: participation - belonging	-1.453	-1.61; -1.31
Difference older: importance - belonging	-0.881	-0.981; -0.791
Without weights (WVS/EVS7)		
Difference younger - older: participation	-0.192	-0.353; -0.037
Difference younger - older: importance	-0.128	-0.176; -0.079
Difference younger - older: belonging	-0.199	-0.311; -0.092
Difference younger: participation - importance	-0.636	-0.777; -0.515
Difference younger: participation - belonging	-1.446	-1.606; -1.304
Difference younger: importance - belonging	-0.81	-0.895; -0.728
Difference older: participation - importance	-0.572	-0.703; -0.454
Difference older: participation - belonging	-1.453	-1.61; -1.31
Difference older: importance - belonging	-0.881	-0.981; -0.791

Note: WVS/EVS7 data. Significance tests using highest (posterior) density intervals, 95%. The first three rows test within the indicators whether younger cohorts are significantly less religious than older cohorts regarding participation, importance, and belonging. The next three rows show test whether the midpoints between indicators for the younger cohorts are significantly different. The last three rows do the same of the older cohorts.

13. Robustness with respect to different prior specifications

We performed a local sensitivity analysis to evaluate the robustness of our findings to different prior specifications (Table 19). Following Gill[3,] we explored less informative priors. As further reducing the degrees of freedom was not advisable, we instead doubled the variance while keeping the degrees of freedom fixed and assessed the impact on the posterior distribution. The results indicate that this adjustment has a negligible effect on the posterior parameters. The CI's are given in Table 20.

Table 19 Results for different priors (doubled variance)

	(1) Baseline			(2) Different priors (doubled Variance)		
	Partici-pation	Impor-tance	Belonging	Partici-pation	Impor-tance	Belonging
Pew						
Midpoint older	-0.37 (0.04)	0.01 (0.03)	1.02 (0.06)	-0.37 (0.04)	0.02 (0.03)	1.01 (0.06)
Midpoint younger	-0.54 (0.04)	-0.15 (0.03)	0.82 (0.05)	-0.53 (0.04)	-0.15 (0.03)	0.81 (0.05)
WVS/EVS7						
Midpoint older	-0.48 (0.07)	0.08 (0.04)	0.96 (0.05)	-0.49 (0.07)	0.08 (0.04)	0.97 (0.05)
Midpoint younger	-0.68 (0.07)	-0.05 (0.04)	0.76 (0.05)	-0.68 (0.07)	-0.04 (0.04)	0.77 (0.05)
WVS/EVS1-7						
Midpoint	-0.56 (0.04)	-0.28 (0.03)	0.74 (0.04)	-0.56 (0.04)	-0.28 (0.03)	0.74 (0.04)

Note: We report posterior midpoints (standard errors).

Table 20 Credibility intervals for differences (different priors, doubled Variance)

Pew:	Difference	CI (95%)
Difference younger-older: participation	-0.162	-0.252; -0.072
Difference younger-older: importance	-0.164	-0.203; -0.125
Difference younger-older: belonging	-0.196	-0.325; -0.08
Difference younger: participation - importance	-0.383	-0.453; -0.315
Difference younger: participation - belonging	-1.346	-1.456; -1.245
Difference younger: importance - belonging	-0.963	-1.052; -0.885
Difference older: participation - importance	-0.385	-0.46; -0.312
Difference older: participation - belonging	-1.38	-1.521; -1.257
Difference older: importance - belonging	-0.995	-1.119; -0.893
WVS/EVS 7:		
Difference younger - older: participation	-0.192	-0.354; -0.036
Difference younger - older: importance	-0.128	-0.176; -0.079
Difference younger - older: belonging	-0.199	-0.312; -0.091
Difference younger: participation - importance	-0.636	-0.778; -0.514
Difference younger: participation - belonging	-1.444	-1.605; -1.301
Difference younger: importance - belonging	-0.809	-0.894; -0.727
Difference older: participation - importance	-0.571	-0.703; -0.454
Difference older: participation - belonging	-1.45	-1.609; -1.307
Difference older: importance - belonging	-0.879	-0.98; -0.79
WVS/EVS 1-7:		
Difference participation - importance	-0.279	-0.355; -0.203
Difference participation - belonging	-1.296	-1.384; -1.213
Difference importance - belonging	-1.018	-1.105; -0.934

Note: Pew and WVS/EVS7 data. Significance tests using highest (posterior) density intervals, 95%. The first three rows test within the indicators whether younger cohorts are significantly less religious than older cohorts regarding participation, importance, and belonging. The next three rows show test whether the midpoints between indicators for the younger cohorts are significantly different. The last three rows do the same of the older cohorts.

14. Finding theoretical time

This paragraph shows how we find an expansion factor lambda permitting to transform relative time into theoretical time in our longitudinal analysis.

We need such a factor lambda since we initially only have regression curves relating time and different measures of religiosity for every country with different levels and slopes but assume that countries with higher levels of religiosity are farther back in the development. The question then arises by what factor these countries are assumed to lag behind. Finding this factor permits us to get a sense of the typical duration of the secular transition.

We initially have
- the relative time position of n countries: D_i,
- their religiosity level Y_i, and
- their change in religiosity over time during the observed period (slope): m.

We now assume an overall trajectory according to a decreasing logistic function (sigmoid function):

$$Y = (1 + e^{a(D - b)})^{-1} \tag{6}$$

Differentiating this equation gives the slope m_0 of the function at every point of D:

$$m_{0i} = (-ae^{(D_i - b)})\,(1 + e^{a(D_i - b)})^{-2} \tag{7}$$

We now have to find a factor λ that transforms m_0 into m.

It turns out that λ is just the inverse of the slope of a regression line through the points (m_{0i}, m_i), constrained to go through the origin.

$$m = \lambda^{-1} m_0 \tag{8}$$

Having found λ we can find theoretical time T with:

$$T = \lambda D \tag{9}$$

It is possible to use this method to find theoretical time for every one of the religiosity indicators, giving every time a somewhat different value. We therefore chose to use a composite measure of religiosity Y to find theoretical time.

SUPPLEMENTARY DISCUSSION

1. A formal model of the link between modernization and the PIB sequence

Our paper does not focus on the causal explanation of the PIB sequence. However, the idea of the PIB sequence suggests a simple economic model that can be interpreted in the sense of a modernization–PIB link. The PIB decline can be seen as an application of a standard economic 2-goods consumption problem.

Let x_r be the amount of religious goods and x_s be the amount of secular goods that an individual intends to consume and let p1 and p2 be their respective prices. The consumer has a fixed income I, which is the budget constraint.

The utility function of the consumer is U(x_r, x_s), which represents the satisfaction or value that the consumer derives from consuming the two goods.

The amount of religious goods x_r is a continuous variable which translates into three discrete, ordered states :

from [0, xra]:	only belonging
from (xra, xrb]:	belonging + importance
from (xrb, + inf):	belonging + importance + participation

Where [a,b] is the closed interval, [a,b) is an interval that includes a but not b, and (a,b] includes b but not a. xra, xrb are two threshold values

The consumer chooses the quantities of goods that maximize their utility subject to income I (= budget constraint). This means to

maximize $U(x_r, y_s)$ subject to $p1x + p2y \leq I$

where x and y are the quantities of goods 1 and 2, respectively.

The maximization happens with:

$$Y = U(x_r, x_s) + \lambda (I - p\, x_r - p\, x_s) \qquad (5)$$

where λ is the Lagrange multiplicator

Differentiating (3) for x_r, x_s, and λ and setting the equations = 0 gives us three equations with three unknowns. We can thus find the optimal amount of x_r and x_s. With the two thresholds we can then find in what state of PIB the individual finds itself.

The theoretical mechanism between modernization and PIB is depicted in Fig. 6.
The slopes of the budget lines are given by the relative prices of secular to religious goods. The intercept on the y-axis is income divided by the price of secular goods. Modernization means, for example, (at constant income), that secular people (who purchase no religious goods) can have more secular goods (because their price decreases). Modernization also means that religious people (at constant income) find themselves in situations in which secular goods become relatively cheaper with respect to religious goods. Modernization will also raise costs of religious goods in terms of cognitive dissonance and decrease the norms prescribing the consumption of religious goods.

In sum, modernization will lead individuals to substitute religious goods with secular goods, while at the same time reaching a higher utility.

This means that a social group may initially be in equilibrium point A, but when modernization ensues, the group will move first to point B, then point C. Doing so, its aggregate religiosity will go through the PIB sequence: it will first drop participation, then importance and will stay with belonging (before dropping even this trait).

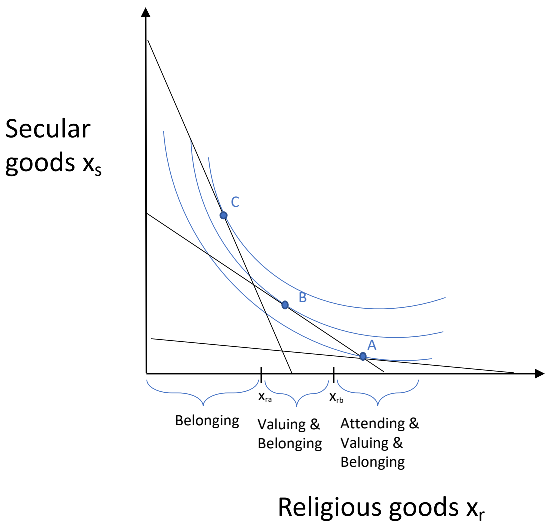

Fig

Fig. 6 The mechanism linking modernization and the PIB sequence

Note that this model goes further than what we claim in our main paper and makes causal predictions. For example, increasing the number of secular goods, or lowering their price, should shift societies along the PIB continuum. As such, the model shows the theoretical background of our argument (a form of secularization theory). Testing this causal model, however, would necessitate a new paper. One of the problems one faces is how to measure the secular goods that stand in competition with religious goods. In many studies HDI or some similar measures are used, but there are many reasons why this may be unsatisfactory.

2. HDI and cohort gaps in religiosity

Our study has demonstrated the existence of the P-I-B sequence but has not attempted to explain it. Such an explanation would have to show the causal effects of explanatory variables on the different timing of the cohort gaps in religiosity indicators. An obvious possible explanatory variable is HDI, but establishing causality will not be an easy task in future studies. We here just give the correlation of HDI, and country secularity (Table 21, Fig. 7), and a visualization of HDI of all countries in the Pew data, together with their cohort gaps for the different indicators (Fig. 8). We use HDI data from the Human Development Reports from 2016 (when analyzing Pew data) and from 2020 (when analyzing WVS7 data). The HDI data can be downloaded at https://hdr.undp.org/data-center/documentation-and-downloads.

Table 21 Correlations of HDI with Country secularity

		Country secularity
Pew data	HDI	.831***
WVS 7 data	HDI	.713***

Note: Country secularity from Pew data refers to studies from 2008-2023; country secularity from WVS/EVS 7 data refers to studies from 2017-2022; GDP/capita and HDI information when correlating with Pew data refers to year 2016; GDP/capita and HDI information when correlating with WVS 7 data refers to year 2020.

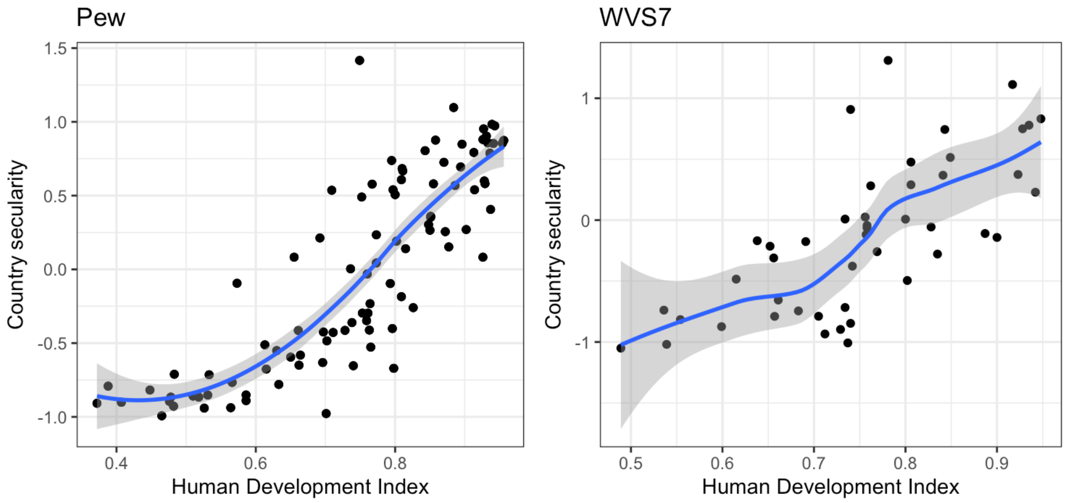

Fig. 7 Human Development Index and Country Secularity in the Pew data and the WVS7 data
Note: Pew data and WVS7 data. The blue line is created with Locally Estimated Scatterplot Smoothing (LOESS) with a smoothing parameter 0.75. The shaded area is a 95% confidence band calculated using the standard errors from the fitted model.

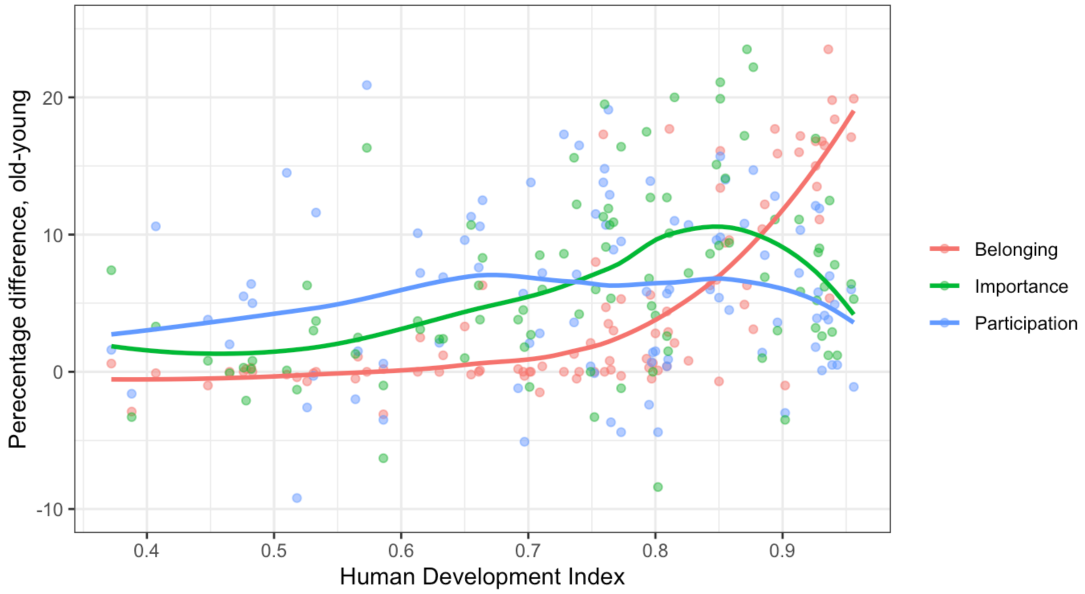

Note: Pew data, HDI information from 2016.

Fig. 8 Human Development and Religiosity-cohort gaps in participation, importance, and belonging in different countries
Note: Pew data. The lines are created with Locally Estimated Scatterplot Smoothing (LOESS) with a smoothing parameter 0.75.

REFERENCES

1. Remizova A, Rudnev M, Davidov E. In Search of a Comparable Measure of Generalized Individual Religiosity in the World Values Survey. *Sociological Methods & Research*, 1-33 (2022).

2. Hackett C, Kramer S, Fahmy D, Marshall J. *The Age Gap in Religion Around the World*, https://www.pewforum.org/2018/06/13/the-age-gap-in-religion-around-the-world/ edn. Pew, Research Center (2018).

3. Gill J. *Bayesian Methods. A Social and Behavioral Sciences Approach. Third Edition*. Taylor and Francis (2015).
