# Supplementary material for: The three stages of religious decline around the world
Source: Nat Commun. 2025 Aug 19;16:7202. doi: 10.1038/s41467-025-62452-z (PMC12365078; doi:10.1038/s41467-025-62452-z)
Supplement: Supplementary file 2 — Reporting Summary [file 41467_2025_62452_MOESM2_ESM.pdf]

nature portfolio

Corresponding author(s): Jörg Stolz

Last updated by author(s): Jun 25, 2025

Reporting Summary

Nature Portfolio wishes to improve the reproducibility of the work that we publish. This form provides structure for consistency and transparency in reporting. For further information on Nature Portfolio policies, see our Editorial Policies and the Editorial Policy Checklist.

Statistics

For all statistical analyses, confirm that the following items are present in the figure legend, table legend, main text, or Methods section.

n/a	Confirmed	
☐	☒	The exact sample size (n) for each experimental group/condition, given as a discrete number and unit of measurement
☐	☒	A statement on whether measurements were taken from distinct samples or whether the same sample was measured repeatedly
☐	☒	The statistical test(s) used AND whether they are one- or two-sided<br>*Only common tests should be described solely by name; describe more complex techniques in the Methods section.*
☐	☒	A description of all covariates tested
☐	☒	A description of any assumptions or corrections, such as tests of normality and adjustment for multiple comparisons
☐	☒	A full description of the statistical parameters including central tendency (e.g. means) or other basic estimates (e.g. regression coefficient) AND variation (e.g. standard deviation) or associated estimates of uncertainty (e.g. confidence intervals)
☒	☐	For null hypothesis testing, the test statistic (e.g. F, t, r) with confidence intervals, effect sizes, degrees of freedom and P value noted<br>*Give P values as exact values whenever suitable.*
☐	☒	For Bayesian analysis, information on the choice of priors and Markov chain Monte Carlo settings
☒	☐	For hierarchical and complex designs, identification of the appropriate level for tests and full reporting of outcomes
☐	☒	Estimates of effect sizes (e.g. Cohen's d, Pearson's r), indicating how they were calculated

Our web collection on statistics for biologists contains articles on many of the points above.

Software and code

Policy information about availability of computer code

Data collection	The study uses data from many different Pew Research Center surveys, all of which are freely available at https://www.pewresearch.org/datasets/. An extract of the relevant data from the Center's surveys that was used in this paper is provided on the Open Science Framework (OSF) at [DOI 10.17605/OSF.IO/VCZTA]. Use of the Pew data complies with the Pew Research Center's Terms and Conditions for data use. This study also uses publicly available data from the World Values Survey and European Values Study, available at https://www.worldvaluessurvey.org/wvs.jsp, accessed in accordance with the website's Terms of Use.
Data analysis	The analysis code and replication materials used in this study are available on the Open Science Framework (OSF) at [DOI 10.17605/OSF.IO/VCZTA].. The repository includes a README file with instructions for reproducing the results.

For manuscripts utilizing custom algorithms or software that are central to the research but not yet described in published literature, software must be made available to editors and reviewers. We strongly encourage code deposition in a community repository (e.g. GitHub). See the Nature Portfolio guidelines for submitting code & software for further information.

Data

Policy information about availability of data

All manuscripts must include a data availability statement. This statement should provide the following information, where applicable:

- Accession codes, unique identifiers, or web links for publicly available datasets
- A description of any restrictions on data availability
- For clinical datasets or third party data, please ensure that the statement adheres to our policy

The study uses data from many different Pew Research Center surveys, all of which are freely available at https://www.pewresearch.org/datasets/. An extract of the relevant data from the Center's surveys that was used in this paper is provided on the Open Science Framework (OSF) at [DOI 10.17605/OSF.IO/VCZTA]. Use of the Pew data complies with the Pew Research Center's Terms and Conditions for data use.
This study also uses publicly available data from the World Values Survey and European Values Study, available at https://www.worldvaluessurvey.org/wvs.jsp, accessed in accordance with the website's Terms of Use.

Research involving human participants, their data, or biological material

Policy information about studies with human participants or human data. See also policy information about sex, gender (identity/presentation), and sexual orientation and race, ethnicity and racism.

Reporting on sex and gender	We use gender as a control variable
Reporting on race, ethnicity, or other socially relevant groupings	We distinguish between countries with different historical religions, on different continents, and belonging to different "cultural clusters". We do not, however, investigate variation of these variables inside the countries. This way of analyzing the data permits illustrating broad trends in a simple way.
Population characteristics	Representative samples of the population in the respective countries, aged 18-70.
Recruitment	Random sampling was effectuated by Pew research center and WVS/EVS.
Ethics oversight	This study is based exclusively on secondary analysis of publicly available, anonymized data from Pew Research Center and the World Values Survey (WVS/EVS). According to the University of Lausanne's research ethics guidelines, the use of such data does not require prior ethics approval. As the data are fully anonymized and collected with informed consent by the original organizations, this research qualifies as exempt from further ethical review.

Note that full information on the approval of the study protocol must also be provided in the manuscript.

Field-specific reporting

Please select the one below that is the best fit for your research. If you are not sure, read the appropriate sections before making your selection.

☐ Life sciences ☒ Behavioural & social sciences ☐ Ecological, evolutionary & environmental sciences

For a reference copy of the document with all sections, see nature.com/documents/nr-reporting-summary-flat.pdf

Behavioural & social sciences study design

All studies must disclose on these points even when the disclosure is negative.

Study description	We use representative cross-sectional datasets for the Pew data analysis and the replication with WVS/EVS 7. We use repeated cross-sectional datasets for the longitudinal replication in 17 countries (with 5 and more measurements for every country).
Research sample	Random samples for the population in the respective countries. The Pew Research Center data and the WVS/EVS data sets are the best international datasets on religion to date.
Sampling strategy	Pew Research Center and WVS/EVS use random sampling
Data collection	Data collection was face-to-face and mixed mode.
Timing	Timing of survey ranges from 1981 to 2023
Data exclusions	We excluded the country Burkina Faso because of too much missing data. In the longitudinal replication we only include countries with five and more measurements in the period between 1981 and 2023.
Non-participation	Response rates vary from survey to survey and are given in the respective publications of Pew Research Center and WVS/EVS.

Randomization	This is an observational study that did not use randomization.

Reporting for specific materials, systems and methods

We require information from authors about some types of materials, experimental systems and methods used in many studies. Here, indicate whether each material, system or method listed is relevant to your study. If you are not sure if a list item applies to your research, read the appropriate section before selecting a response.

Materials & experimental systems

n/a	Involved in the study
☒	☐ Antibodies
☒	☐ Eukaryotic cell lines
☒	☐ Palaeontology and archaeology
☒	☐ Animals and other organisms
☒	☐ Clinical data
☒	☐ Dual use research of concern
☒	☐ Plants

Methods

n/a	Involved in the study
☒	☐ ChIP-seq
☒	☐ Flow cytometry
☒	☐ MRI-based neuroimaging

Plants

Seed stocks	n/a
Novel plant genotypes	n/a
Authentication	n/a
